# Tyrosine kinases inhibition by Imatinib slows progression in chronic anti-thy1 glomerulosclerosis of the rat

**DOI:** 10.1186/1471-2369-14-223

**Published:** 2013-10-14

**Authors:** Yingrui Wang-Rosenke, Dymtro Khadzhynov, Tanja Loof, Alice Mika, Hiroshi Kawachi, Hans-H Neumayer, Harm Peters

**Affiliations:** 1 Department of Nephrology and Center of Cardiovascular Research, Campus Charité Mitte, Charité University Medicine Berlin, Charitéplatz 1, Berlin D-10117, Germany; 2 Department of Cell Biology and Institute of Nephrology, Niigata University Graduate School of Medical and Dental Sciences, Niigata Japan

**Keywords:** Imatinib, TGF-β1, Platelet-derived growth factor, Fibrosis, Mesangioproliferative nephropathy

## Abstract

**Background:**

Chronic progressive mesangioproliferative nephropathy represents a major cause of end-stage renal disease worldwide. Until now, effective approaches to stop or even slow its progression are limited. We tested the effects of an inhibitor of PDGF receptor, abl and c-kit tyrosine kinases, Imatinib, in a chronic progressive model of mesangioproliferative glomerulosclerosis.

**Methods:**

Anti-thy1 glomerulosclerosis was induced by injection of anti-thy1 antibody into uninephrectomized Wistar rats. One week after disease induction, according to the degree of proteinuria, animals were stratified and assigned to chronic glomerulosclerosis (cGS) and cGS plus Imatinib (10 mg/kg body weight/day). In week 20, renoprotective actions of Imatinib were analyzed by a set of functional, histological and molecular biological parameters.

**Results:**

Untreated cGS rats showed elevation of systolic blood pressure and marked progression in proteinuria, renal fibrosis, cell infiltration, cell proliferation and function lost. Administration of Imatinib went along significantly with lower systolic blood pressure (−10 mmHg) and proteinuria (−33%). Imatinib administration was paralled by significant reductions in tubulointerstitial accumulation of matrix proteins (−44%), collagen I deposition (−86%), expression of TGF-beta1 (−30%), production of fibronectin (−23%), myofibroblast differentiation (−87%), macrophage infiltration (−36%) and cell proliferation (−45%), respectively. In comparison with untreated cGS animals, Imatinib therapy lowered also blood creatinine (−41%) and blood urea concentrations (−36%) and improved creatinine clearance (+25%). Glomerular fibrotic changes were lowered moderately by Imatinib.

**Conclusions:**

Therapy with Imatinib limits the progressive course of chronic anti-thy1 glomerulosclerosis towards tubulointerstitial fibrosis and renal insufficiency. This was paralleled by direct and indirect sign of TGF-β1 and PDGF inhibition. The findings suggest that the pharmacological principal of inhibition of tyrosine kinases with drugs such as Imatinib might serve as approach for limiting progression of human mesangioproliferative glomerulosclerosis.

## Background

IgA nephropathy, a mesangial proliferative glomerulonephritis, is the most common primary glomerulonephritis worldwide, and as many as 20-30% of patients with IgA nephropathy progress to end-stage renal failure after 20–25 years [1-4]. It is characterized by in the beginning phase with expansion of glomerular mesangial matrix and mesangial cell proliferation, and a subsequent progression phase with glomerulosclerosis, tubulointerstitial fibrosis and ongoing loss in renal function, which is a hallmark of many chronic glomerulonephritis [5]. The progress from glomerulonephritis to end-stage renal disease and the need for renal replacement therapy can even be seen when the initial glomerulonephritic phase has been resolved, suggesting a self-perpetuated and intrarenal mechanism is operating during the disease progression.

Data from numerous studies of experimental and human diseases have shown that persistent overexpression of the cytokines transforming growth factor-β (TGF-β) and platelet-derived growth factor (PDGF) are key markers and mediators of tissue matrix accumulation and cell proliferation in progressive renal disease [6,7]. Prominent characteristics of chronic renal disease are expansion of extracellular matrix expansion, renal cell proliferation and cell infiltration as well as the appearance of “activated” fibroblasts characterized by α-smooth muscle actin (SMA) [8,9]. The origin of these myofibroblasts is unclear but may result from growth factor-mediated differentiation of resident mesenchymal cells or recruitment of microvascular pericytes. Recent evidence has suggested that TGF-β induces the differentiation of resident mesenchymal cells to myofibroblast and PDGF appears to affect pericyte differentiation and recruitment [10,11]. In turn, specific inhibitions of TGF-β and PDGF pathways and action have increasingly been explored as therapeutic approaches for progressive renal disease.

Imatinib mesylate (formerly STI 571; Glivec/Gleevec) inhibits Abelson (c-Abl)- and c-kit kinases, as well as PDGF receptor α and β [12]. It has been already used clinically in treatment of diseases with *abl-* and *c-kit* kinases overexpression, such as gastrointestinal stromal tumors and chronic myeloid leukemia [13]. In vitro studies have demonstrated that Bcr-Abl might be a down-stream mediator of TGF-β signalling in fibroblasts [14]. Imatinib has shown anti-fibrotic effects in different animal models with organ fibrosis, including acute anti-thy1 glomerulonephritis of the rat [15].

In this study, we examined the effects of Imatinib in a model of progressive mesangioprolifertive glomerulosclerosis. The novel finding of this study is that expands from the acute anti-thy1 glomerulonephritis into a anti-thy1-induced chronic-progressive glomerulosclerosis model of human mesangioproliferative nephropathy as a leading cause of end-stage kidney disease worldwide. In this model, injection of high dose anti-thy1 antibody into uninephrectomized rats leads to a brief period of acute mesangioproliferative glomerulonephritis which is followed by an autonomous progression towards glomerulosclerosis, tubulointerstitial fibrosis and renal insufficiency over months. An acute, reversible, and 4-week course of the disease occurs when a relatively low dose of anti-thy1 antibody is injected into animals with two kidneys, where the overproduction of TGF-β is transient [16].

Treatment with Imatinib was started 1 week after antibody injection. Effects of Imatinib treatment on proteinuria, blood pressure, glomerular and tubulointerstitial fibrosis, molecular markers of TGF-β and PDGF pathways and renal function were determined in week 20 after disease induction.

## Methods

### Materials

All materials, chemicals and cell culture media used, if not stated differently, were purchased from Sigma Chemical-Aldrich Co. (Taufkirchen, Germany).

### Animals and model of anti-thy1-induced chronic-progressive glomerulosclerosis

Male Wistar rats (150–180 g, Charles River, Sulzfeld, Germany) were caged in a constant temperature room with a 12 h dark/12 h light cycle and fed a normal protein diet (22.5% protein, Altromin, Lage, Germany) for at least 3 days before the start of the experiment to allow equilibration. The animals were visited daily, and the consumption of food and drinking water and body weight were monitored every 2–3 days.

Anti-thy1-induced chronic-progressvie glomerulosclerosis (cGS) was induced by intravenously injecting the monoclonal antibody mAb 1-22-3 (5 mg/kg body weight in phosphate-buffered saline [PBS], pH =7.4) three days after uni-nephrectomy as previously described [17]. mAb 1-22-3 antibody binds to a thy1-like antigen on mesangial cells and causes a fast complement- and NO-dependent mesangial cell lysis within the next 24 h [18]. The uninephrectomy being performed before anti-thy1 antibody injection is related to the chronic progression of cGS, since the glomerular disease resolves over approximately 4 weeks in animals with two kidneys. Control animals with and without uninephrectomy were injected with equal volumes of PBS only.

Animal care and treatment were in conformity with the ARRIVE (Animal Research: Reporting In Vivo Experiments) guidelines being developed by the NC3Rs and approved by local authorities (animal experiments, Landesamt für Arbeitsschutz, Gesundheitsschutz und technische Sicherheit Berlin).

### Study groups and design

Nonnephrectomized animals injected with PBS (2-K Control, n = 4) and uninephrectomized animals injected with PBS (1-K Control, n = 4) served as controls. On the basis of the actual 24-h proteinuria achieved one week after anti-thy1 antibody injection, the diseased animals were stratified assigned to the uni-nephrectomized, anti-thy1-injected animals, no treatment (cGS, n = 11) and uni-nephrectomized, anti-thy1-injected animals treated with Imatinib (cGS + Imatinib, n = 11) groups.

Treatments were started seven days after antibody injection, to avoid interference with the induction of disease by anti-thy1 antibody. Imatinib is chemically designated as 4-[(4-methyl-1-piperazinyl)medthyl]-N-[4-methyl-3-[[4-(3-pyridinyl)-2-pyrimidinyl]amino]-phenyl] benzamide methanesulfonate [12]. Imatinib is designed to specifically interact with the adenosine triphosphate (ATP)-binding site of protein tyrosine kinases, a selective inhibitor of the tyrosine kinases Bcr-Abl, PDGF receptors, and c-kit [12]. It was given with the food at a daily dose of 10 mg/kg body weight. The dose was chosen on the basis of previous reports showing that this dose reduced diabetic nephropathy progression in rats [19]. The drug-containing food was produced by mixing Imatinib mesylate with the flour of the standard rat chow (22.5% protein, A1311, Altromin), and water was added to form pellets which were subsequently given to the animals after being air-dried [17].

In week 20, i.e. after 19 weeks of treatment, the actions of tyrosine kinases signal transduction inhibition by Imatinib on proteinuria, systolic blood pressure, matrix protein expansion, macrophage infiltration, cell proliferation and kidney function were determined. Glomerular and tubulointerstitial changes were analyzed separately. Glomeruli were isolated by a graded sieving technique. Since the renal cortex consists mainly of tubulointerstitial tissue (>95%), it was used as representative for the tubulointerstitium. Analysis of fibrosis involved a computer-based histological calculation of the matrix and collagen I actually accumulated as well as molecular analysis of the expression of the key fibrosis marker and mediator TGF-β1, the matrix protein fibronectin which indicates matrix protein synthesis, and the tissue inhibitor of metalloproteinase-1 (TIMP-1) as a marker of matrix protein degradation. Tubulointerstitial and glomerular myofibroblast differentiation, macrophage infiltration and cell proliferation were analyzed by immunohistochemistry using an α-SMA-, ED1- or a Proliferating-Cell-Nuclear-Antigen (PCNA) -antibody, respectively. In addition, blood creatinine and urea concentrations, and calculated creatinine clearance served as markers of renal function.

### Blood pressure and proteinuria

Systolic blood pressure was assessed in weeks 10 and 20 in trained conscious animals using tail cuff plethysmography as previously described [16]. 1, 8 and 19 weeks after disease induction, animals were housed individually in metabolic cages for 24-hour urine collection. Urinary protein was determined by a pyrogallol red method [16] and is expressed as mg protein/24 h.

### Sacrifice

The experiments were terminated by anesthetizing animals with 0.1 mg ketanest/ 0.01 mg xylazin per 100 g body weight (Ketamin 10%, WDT, Garbsen, Germany; Rompun 2%, Bayer Vital GmbH, Leverkusen; Germany). Following laparotomy, blood was drawn from the abdominal aorta into EDTA-coated tubes and kidneys were subsequently perfused with 40 mL ice-cold PBS. Materials and tissues were subsequently processed as described in the following sections.

### Renal function analysis

Spectrometrical enzyme-based assays were used to measure plasma and urine creatinine and plasma urea. Glomerular filtration rate (GFR) was calculated subsequently on the basis of the corresponding urine volume and is expressed as ml per minute per 100 g body weight [16].

### Histology and immunohistochemistry

All microscopic examinations were performed in a blinded fashion as previously reported [20]. For histological examination, cortical tissue was fixed in Carnoy’s solution. Three-μm sections of paraffin-embedded tissue were stained with periodic acid-Schiff (PAS) to analyze tubulointerstitial and glomerular fibrosis by a computer-based morphometric analysis. Renal sections were examined on a Leica DM LB2 light microscope (Leica Microsystems, Wetzlar, Germany) connected to a PL-A662 video camera and the Axiovision 2.05 image analysis system (both Karl Zeiss Vision GmbH, München, Germany) using a 10 × 10 orthographic grid overlaid on digital images. The relative degree of tubulointerstitial fibrotic lesions, i.e. matrix deposition, tubular atrophy and dilation was calculated in 15 randomly-selected cortical areas per animal observed at ×200 magnification. It is expressed as percentage of the area affected in relation to the total area analyzed. Glomerular matrix expansion was evaluated by calculating the relative degree of the mesangial matrix-occupying area (in percent) of 15 glomeruli from each rat.

Renal myofibroblast differentiation, macrophage infiltration and cell proliferation were analyzed on paraffin-embedded tissues incubated with a primary mouse anti-α-SMA or ED1 antibody (Serotec, Oxford, UK) in conjunction with a standard APAAP technique (DakoCytomation, Hamburg, Germany), and using a primary mouse anti-PCNA-antibody (DakoCytomation) and a secondary goat anti-mouse antibody coupled with the Envision™ staining system (DakoCytomation), as previously described [16,18]. Immunohistochemistry for detecting type I collagen was performed by using goat anti-type I collagen primary antibody (Southern Biotech, distributed by Biozol, Eching, Germany). As a secondary antibody, horseradish peroxidase-conjugated rabbit anti-goat antibody was used and visualized with AEC reagent (all DakoCytomation, Glostrup, Denmark). Renal collagen I deposition, myofibroblast differentiation, macrophage infiltration and cell proliferation evaluated by collagen and α-SMA positive staining, ED1- and PCNA-positive cells, respectively in at least 15 glomerular sections and at least 15 randomly-selected cortical areas from each rat observed at ×200 magnification. Collagen I deposition and myofibroblast were expressed as percentage per area by applying the histomorphometric computer-based Axiovision 4.1 image analysis system.

### Glomerular and cortical protein expression of TGF-β1, fibronectin and TIMP-1

Glomeruli from individual rats were isolated by a graded sieving technique (160, 125 and 71 μm mesh metal sieves), as described previously [16]. For cultures of renal cortical tissue, a piece of cortical tissue was weighed and minced extensively with a razor blade [16]. Glomeruli or cortical tissues were suspended in DMEM supplemented with 0.1 U/mL insulin, 100 U/mL penicillin and 100 μg/mL streptomycin at a density of 2000 glomeruli/mL and 10 mg/mL, respectively. After 48 h incubation at 37°C/5% CO_2_, supernatants were harvested and stored at −20°C until further analysis. TGF-β1 content of culture supernatant was measured after acid activation, using a commercially available enzyme-linked immunosorbent assay (ELISA) kit (R&D Systems, Wiesbaden, Germany) according to the manufacturer’s instructions. TIMP-1 levels were analyzed using another commercially available ELISA kit (R&D Systems, Wiesbaden, Germany). Fibronectin was measured with a modified competitive ELISA, according to published methods [18]. Three samples from each rat were analyzed.

### Quantitation of tubulointerstitial mRNA expression

Cortical total RNA was extracted with Trizol™ reagent (Gibco BRL, Berlin, Germany) according to the manufacturer’s instructions. The mRNA expression was determined by a “two-step” reverse transcription-polymerase chain reaction (RT-PCR) [18]. A cDNA copy was created with reverse transcriptase from RNA PCR Core kit (Roche, Applied Biosystems, New Jersey, USA). Real-time PCR was performed using the LightCycler System and SYBR Green I as dsDNA binding dye (Roche Diagnostics GmbH, Mannheim). The following primer pairs were used: PDGF-A (sense 5′-GCCTTGGAGACAAACCTGAG-3′ and antisense 5′-AAATGACCGTCCTGGTCTTG-3′), -B (sense 5′- ACACCTCAAACTCGGGTGAC-3′ and antisense 5′-TCAGTGCCTTCTTGTCATGG-3′), -C (sense 5′- CCAAGAAATACGGTGCTGGT-3′ and antisense 5′-CATCACTGGGCTCCTCAACT-3′), -D (sense 5′- CCCAGGAGAAAACACGGATA-3′ and antisense 5′-CCTTATGGCCACACCATCTT-3′) and receptor-α (sense 5′-AGAAGATTGTGCCGCTGAGT-3′ and antisense 5′-TCTTCGTTTCTGATTTCCAC-3′) and –β (sense 5′-ACACATCAAATACGCGGACA-3′ and antisense 5′-GAGCACTGGTGAGTCGTTGA-3′), and glyceraldehyde-3-phosphate dehydrogenase (GAPDH) (sense 5′- CCATCTTCCAGGAGCGAGAT-3′ and antisense 5′-GATGACCTTGCCCACAGCCT-3′) as housekeeping gene were used as previously described [21]. For analysis, a relative quantification method was used as previously described [22].

### Statistical analysis

The results are expressed as mean ± standard deviation (SD). Our data were not normally distributed. Statistical analysis between groups was performed by Kruskal-Wallis and subsequent Mann–Whitney *U-* testing. A p-value lower than 0.05 was considered significant.

## Results

### Body weight, food and drug intake

At the end of the experiment, animals’ mean body weights were 601 ± 68 g in the 2-K-Control, 544 ± 34 g in the 1-K Control, 523 ± 40 g in the cGS and 497 ± 30 g in the cGS + Imatinib group, respectively (p = NS between both cGS groups). Mean food and water intakes did not significantly differ between the groups throughout the experiment (data not shown).

### Proteinuria, blood pressure and renal function

**Figure 1:**
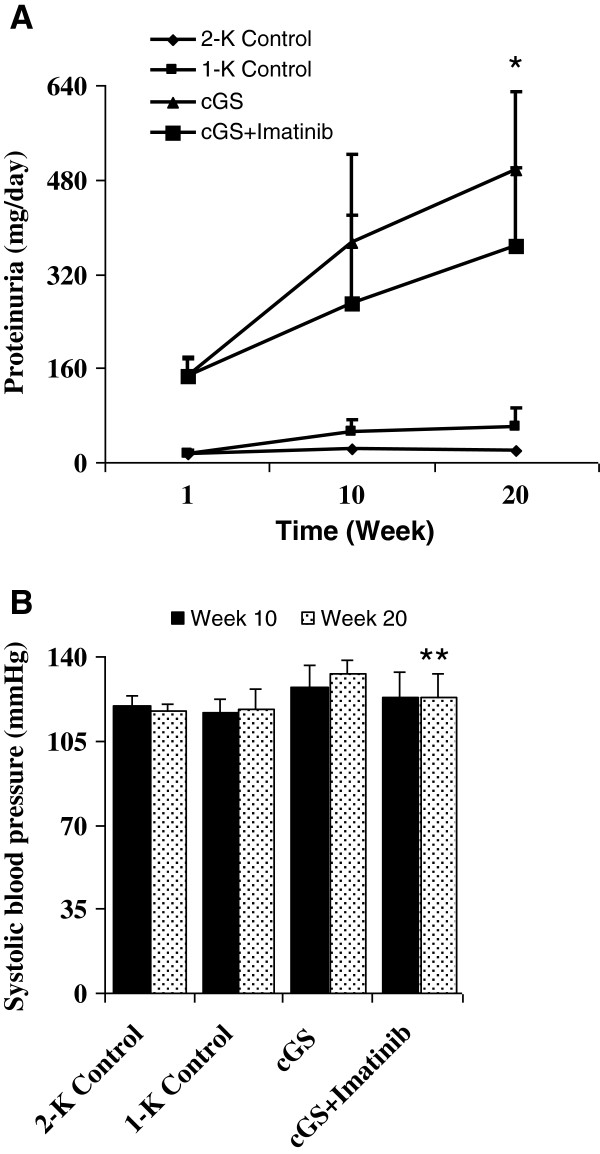
**Effects of Imatinib on time course of proteinuria (A) and systolic blood pressure after 10 and 20 weeks (B) in chronic anti-thy1 glomerulosclerosis (cGS).** Administration of Imatinib was started 7 days after injection of anti-thy1 antibody into uni-nephrectomized rats (cGS + Imatinib). Nonnephritic animals with (1-K Control) and without uninephrectomy (2-K Control) received an injection with similar volumes of PBS. Blood pressure was measured in conscious animals using a tail cuff method. Urine was collected for 24 h at the indicated times, using metabolic cages. (*p < 0.05 and **p < 0.01 vs. cGS).

Before the start of therapy, nephritic animals were stratified to start with equal levels of pre-treatment proteinuria in the two diseased groups (Figure 1A) (cGS: 147 ± 30 mg/d, cGS + Imatinib: 146 ± 30 mg/d, P = NS). Urinary protein loss increased gradually in untreated diseased animal groups during the experiment (week 10: 374 ± 149 mg/d and week 20: 495 ± 133 mg/d, p < 0.01 vs. Controls). Administration of Imatinib slowed the deterioration of urinary protein excretion. In week 20, proteinuria was significantly lower in the Imatinib-treated animals (cGS + Imatinib 366 ± 133 mg/d, p < 0.05 vs. cGS).

As shown in Figure 1B, systolic blood pressure was increased slightly during the disease progression in the anti-thy1-induced chronic glomerulosclerosis model (week 10: 128 ± 7 mmHg and week 20: 133 ± 7 mmHg, p < 0.05 vs. Controls). In week 20, treatment with Imatinib reduced systolic blood pressure significantly (123 ± 10 mmHg, p < 0.01 vs. cGS).

As shown in Table 1, animals with chronic anti-thy1 glomerulosclerosis showed significant increases in blood creatinine (2.43 ± 2.12 mg/dL) and urea concentrations (232 ± 182 mg/dL) and decrease in creatinine clearances (0.24 ± 0.16 mL/min) (all P < 0.01 vs. 1-K Control), indicating chronic renal insufficiency. Therapy with Imatinib lowered plasma creatinine levels (−41%, 1.42 ± 1.13 mg/dL) and urea levels (−36%, 148 ± 109 mg/dL), and preserved creatinine clearances (+25%, 0.32 ± 0.16 mL/min), although they didn’t reach significance.

**Table 1:** Effects of Imatinib on markers of renal function 20 weeks after induction of chronic anti-thy1 glomerulosclerosis (cGS)

**Group/parameter**	**Creatinine (mg/dL)**	**Urea (mg/dL)**	**GFR/100 g body weight mL/min**
2-K Control	0.41 ± 0.12	42 ± 10	0.68 ± 0.04
1-K Control	0.49 ± 0.04	44 ± 6	0.49 ± 0.08
cGS	2.43 ± 2.12**	232 ± 182**	0.24 ± 0.16**
cGS + Imatinib	1.42 ± 1.13	148 ± 109	0.32 ± 0.16

Administration of Imatinib was started 7 days after injection of anti-thy1 antibody into uni-nephrectomized rats (cGS + Imatinib). Nonnephritic animals with (1-K Control) and without uninephrectomy (2-K Control) received an injection with similar volumes of PBS. GFR is glomerular filtration rate. Plasma creatinine and urea levels and creatinine clearance served as markers of excretory kidney function. (**p < 0.01 vs Controls).

**Figure 2:**
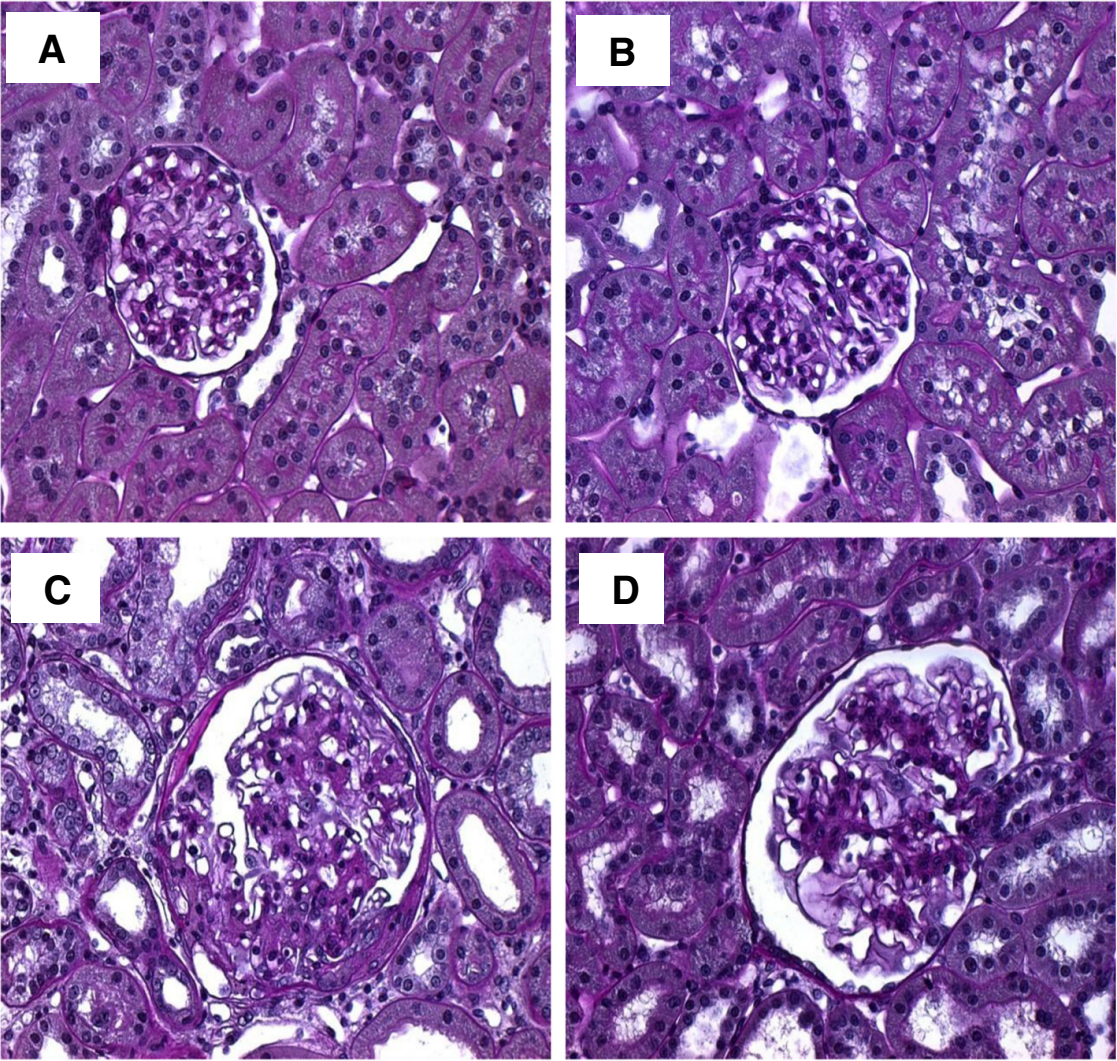
**Renal histology 20 weeks after induction of chronic anti-thy1 glomerulosclerosis (cGS).** Administration of Imatinib was started 7 days after injection of anti-thy1 antibody into uni-nephrectomized rats (cGS + Imatinib). Nonnephritic animals with (1-K Control) and without uninephrectomy (2-K Control) received an injection with similar volumes of PBS. Shown are characteristic periodic acid-schiff (PAS)-stained renal sections from a normal control animal **(A)**, an animal with uninephrectomie **(B)**, an animal with anti-thy1-induced cGS without **(C)** and with Imatinib **(D)** treatment. Magnification ×400.

**Figure 3:**
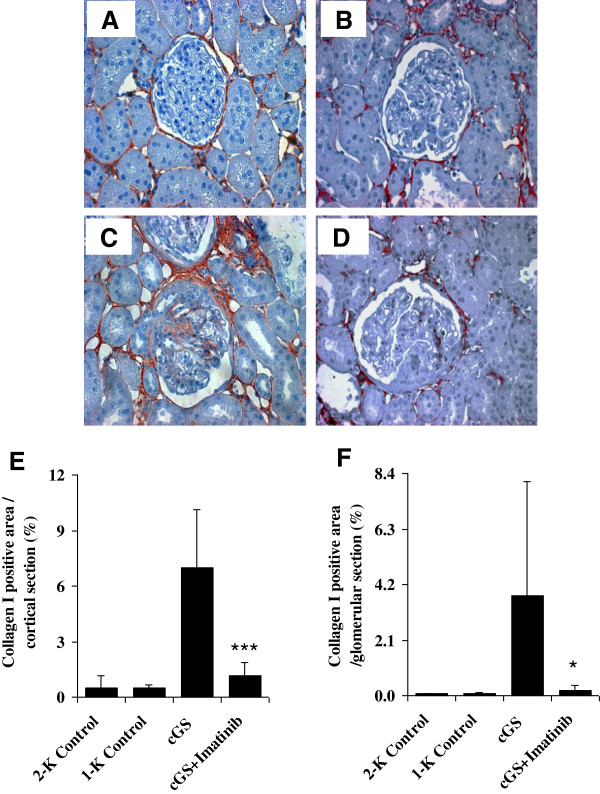
**Glomerular and tubulointerstitial expression of collagen I 20 weeks after induction of chronic anti-thy1 glomerulosclerosis (cGS).** Representative photographs of immunhistochemically stained sections show a normal control animal **(A)**, an animal with uninephrectomy **(B)**, an animal with anti-thy1-induced cGS without **(C)** and with Imatinib **(D)** treatment. Graphs depict the percentages of collagen I-positive staining of at least 15 randomized tubulointerstitial **(E)** and glomerular sections **(F)** obtained by computer-based histomorphometry. Administration of Imatinib was started 7 days after injection of anti-thy1 antibody into uni-nephrectomized rats (cGS + Imatinib). Nonnephritic animals with (1-K Control) and without uninephrectomy (2-K Control) received an injection with similar volumes of PBS. Magnification ×200. (*P < 0.05 and ***P < 0.001 vs. cGS).

The histological photographs in Figures 2 and 3 provide characteristic overviews on the effects of Imatinib treatment on renal matrix accumulation in anti-thy1-induced chronic glomerulosclerosis. The most pronounced actions of Imatinib were seen in the tubulointerstitial compartment.

### Tubulointerstitial matrix accumulation

**Figure 4:**
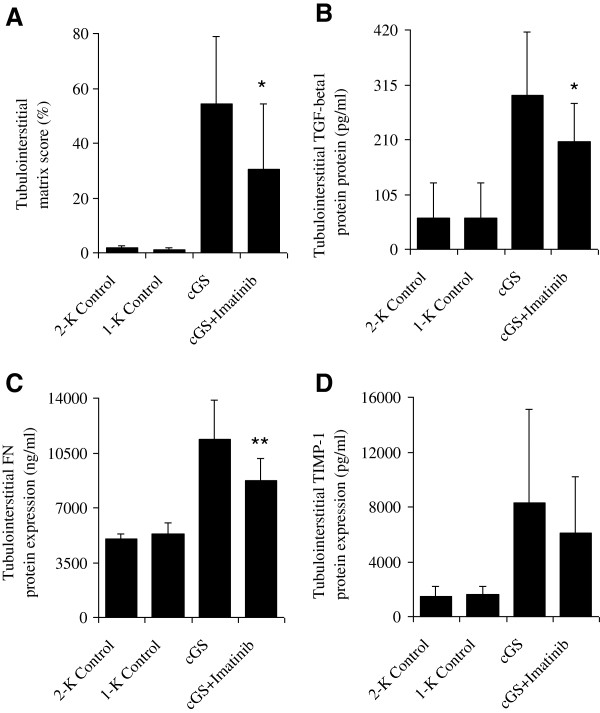
**Effects of Imatinib on tubulointerstitial matrix protein expression 20 weeks after induction of chronic anti-thy1 glomerulosclerosis (cGS).** Shown are tubulointerstitial matrix accumulation **(A)** and protein expression of TGF-β1 **(B)**, fibronectin **(C)** and TIMP-1 **(D)**. Administration of Imatinib was started 7 days after injection of anti-thy1 antibody into uni-nephrectomized rats (cGS + Imatinib). Nonnephritic animals with (1-K Control) and without uninephrectomy (2-K Control) received an injection with similar volumes of PBS. The relative degree of matrix accumulation was calculated by computer-based morphometric analysis. Matrix protein production was determined in extensively minced individual cortical tissues cultured at a density of 10 mg/mL for 48 h (*p < 0.05 and **p < 0.01 vs. cGS).

As shown in Figures 4 and 3, there was a marked increase in histological tubulointerstitial matrix score (54 ± 25%) and collagen I deposition (7 ± 3%), and protein expression of TGF-β1 (294 ± 121 pg/mL), fibronectin (11392 ± 2498 ng/mL) and TIMP-1 (8320 ± 6812 pg/mL) when compared to non-nephritic control animals (all p < 0.01 vs. Control) in week 20 after disease induction. In turn, treatment with Imatinib reduced histological tubulointerstitial matrix accumulation (−44%*, 30 ± 24%) and collagen I deposition (−86%***, 1.1 ± 0.7%), and protein expressions of TGF-β1 (−30%*, 206 ± 73 pg/mL), fibronectin (−23%**, 8744 ± 1383 ng/mL) and TIMP-1 (−26%, 6120 ± 4086 pg/mL), respectively (*** p < 0.001, ** p < 0.01 and *p < 0.05 vs. cGS) (Figures 4 and 3).

### Glomerular matrix accumulation

As shown in Figure 3 and Table 2, glomerular matrix protein accumulation was characterized by an increase in histological matrix score (56 ± 17%), collagen I deposition (3.8 ± 4.3%), and protein expression of TGF-β1 (101 ± 76 pg/mL) and fibronectin (5625 ± 1570 ng/mL) at the end of the experiment (all p < 0.01 vs. 1-K Control). Administration of Imatinib lowered histological matrix accumulation (−13%, 49 ± 17%), collagen I deposition (−95%, 0.2 ± 0.2%, p < 0.05 vs. cGS), TGF-β1 (−17%, 84 ± 52 pg/mL) and fibronectin (−13%, 4879 ± 1387 ng/mL).

**Table 2:** Effects of Imatinib on glomerular matrix protein expression 20 weeks after induction of chronic anti-thy1 glomerulosclerosis (cGS)

**Group/parameter**	**Glomerular matrix score (%)**	**Glomerular TGF-βprotein expression (pg/ml)**	**Glomerular fibronectin protein expression (pg/ml)**
2-K Control	21 ± 4	22 ± 22	2600 ± 182
1-K Control	24 ± 4	28 ± 30	2695 ± 356
cGS	56 ± 17**	101 ± 76**	5625 ± 1570**
cGS + Imatinib	49 ± 17	84 ± 52	4879 ± 1387

Shown are glomerular matrix accumulation and protein expression of TGF-β1 and fibronectin. Administration of Imatinib was started 7 days after injection of anti-thy1 antibody into uni-nephrectomized rats (cGS + Imatinib). Nonnephritic animals with (1-K Control) and without uninephrectomy (2-K Control) received an injection with similar volumes of PBS. Matrix expansion was scored on PAS-stained slides (%). Glomeruli were harvested from individual animals and cultured at a density of 2000 per mL for 48 h. (**p < 0.01 vs. Controls).

### Renal myofibroblast differentiation

**Figure 5:**
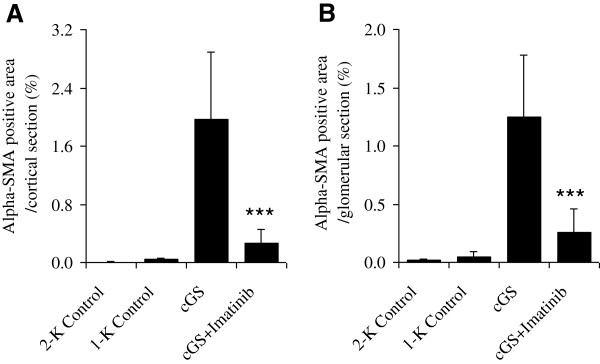
**Effects of Imatinib on tubulointerstitial (A) and glomerular (B) myofibroblast differentiation 20 weeks after induction of chronic anti-thy1 glomerulosclerosis (cGS).** Administration of Imatinib was started 7 days after injection of anti-thy1 antibody into uni-nephrectomized rats (cGS + Imatinib). Nonnephritic animals with (1-K Control) and without uninephrectomy (2-K Control) received an injection with similar volumes of PBS. Analysis was performed using a primary α-SMA-antibody for myofibroblast. Data are expressed as percentage of α-SMA-positive expression per cortical section observed at ×200 magnification and per glomerular cross section (***p < 0.001 vs. cGS).

As shown in Figure 5, uninephrectomized, nonnephritic animals showed a low number of glomerular and tubulointerstitial α-SMA expressing myofibroblasts (1-K Control: 0.048 ± 0.048% and 0.038 ± 0.016%). In contrast, rats with progressive anti-thy1-induced glomerulosclerosis expressed marked increases in glomerular and tubulointerstitial α-SMA expression (1.25 ± 0.53% and 1.96 ± 0.93%, p < 0.001 vs. 1-K Controls). The number of α-SMA-positive myofibroblasts in the glomeruli and tubulointerstitium was reduced by −79% (0.26 ± 0.2%) and −87% (0.26 ± 0.2%) after Imatinib treatment (p < 0.001 vs. cGS), respectively.

### Renal macrophage infiltration and cell proliferation

**Figure 6:**
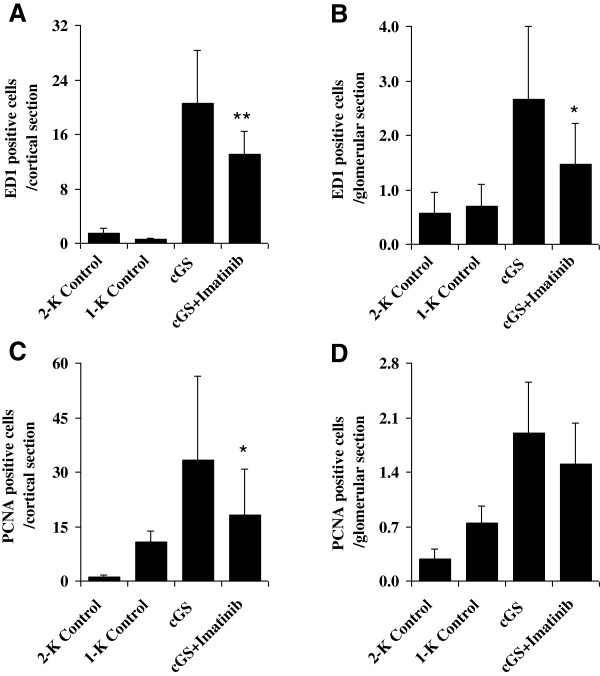
**Effects of Imatinib on tubulointerstitial and glomerular macrophage infiltration (A, B) and cell proliferation (C, D) 20 weeks after induction of chronic anti-thy1 glomerulosclerosis (cGS).** Administration of Imatinib was started 7 days after injection of anti-thy1 antibody into uni-nephrectomized rats (cGS + Imatinib). Nonnephritic animals with (1-K Control) and without uninephrectomy (2-K Control) received an injection with similar volumes of PBS. Analysis was performed using a primary ED1-antibody for macrophages and a primary PCNA-antibody for proliferating cells. Data are expressed as cells per cortical section observed at ×200 magnification and per glomerular cross section (*p < 0.05 and **p < 0.01 vs. cGS).

Chronic anti-thy1-induced glomerulosclerosis was accompanied by prominent renal macrophage infiltration and cell proliferation, both in the tubulointerstitial and glomerular compartment. As shown in Figure 6, in the group with progressive anti-thy1-induced glomerulosclerosis, ED1-positive cells indicating macrophages were increased 32-fold at the tubulointerstitial level (20 ± 7.8 ED1 positive cells/cortical section), and 4-fold at the glomerular level (2.6 ± 1.3 ED1 positive cells/glomerular section), while PCNA-positive tubulointerstitial cells indicating cell proliferation were elevated by 4-fold (33 ± 23 PCNA positive cells/cortical section) and PCNA-positive glomerular cells by 2-fold (1.9 ± 0.6 PCNA positive cells/glomerular section), respectively (both p < 0.05 vs. Control). Treatment with Imatinib reduced both tubulointerstitial and glomerular infiltration with macrophages (−36%**, 13 ± 3.4 ED1 positive cells/cortical section and −45%*, 1.5 ± 0.7 ED1 positive cells/glomerular section) and tubulointerstitial and glomerular proliferation of cells (−45%*, 18 ± 13 PCNA positive cells/cortical section and −21%, 1.5 ± 0.5 PCNA positive cells/glomerular section)) (**p < 0.01 and *p < 0.05 vs. cGS).

### Tubulointerstitial mRNA expression of PDGF signal transduction

As shown in Table 3, compared to controls, the induction of chronic progressive anti-thy1 induced glomerulosclerosis increased mRNA expression of PDGF-A, B, C and D as well as PDFG receptor-α and receptor-β. Treatment with Imatinib had no significant effect on the mRNA expression of PDGF signal transduction when compared to the untreated cGS group.

**Table 3:** Effects of Imatinib on tubulointerstitial mRNA expressions of PDGF signal transduction 20 weeks after induction of chronic anti-thy1 glomerulosclerosis (cGS)

**Group/parameter**	**PDGF-A (%)**	**PDGF-B (%)**	**PDGF-C (%)**	**PDGF-D (%)**	**PDGF-α (%)**	**PDGF-β(%)**
2-K Control	98 ± 22	111 ± 56	101 ± 50	126 ± 42	106 ± 42	118 ± 74
1-K Control	90 ± 4	114 ± 50	89 ± 18	102 ± 50	91 ± 66	75 ± 22
cGS	306 ± 318*	263 ± 172*	83 ± 66	265 ± 186*	383 ± 222**	307 ± 179**
cGS + Imatinib	172 ± 56**	224 ± 96*	84 ± 23	261 ± 126**	355 ± 186**	305 ± 156**

Shown are tubulointerstitial mRNA expressions of PDGF-A, -B, -C, -D and receptor-alpha and –beta. Administration of Imatinib was started 7 days after injection of anti-thy1 antibody into uni-nephrectomized rats (cGS + Imatinib). Nonnephritic animals with (1-K Control) and without uninephrectomy (2-K Control) received an injection with similar volumes of PBS. mRNA was analyzed by a real-time polymerase chain reaction (PCR) method using glyceraldehydes-3-phosphate dehydrogenase (GAPDH) as housekeeping gene. (**p < 0.01 and *p < 0.05 vs. Controls).

Taken together, the present study demonstrates that inhibition of tyrosine kinases signal transduction limits the progressive course of anti-thy1-induced chronic renal disease towards glomerulosclerosis, tubulointerstitial fibrosis and renal insufficiency. Renoprotection by Imatinib was associated with reductions in renal matrix accumulation, TGF-β overproduction, myofibroblast differentiation, cell proliferation and macrophage infiltration.

## Discussion

Tyrosine kinases regulate a wide variety of normal cell processes, including metabolism, growth, differentiation and apoptosis. Pathological activation of tyrosine kinases may drive carcinogenesis, vascular remodeling and fibrogenesis [23-25]. Imatinib was initially developed for its selective action against the Bcr-Abl fusion protein, a key driver of chronic myeloid leukemia [12]. The activities of PDGF and c-Kit tyrosine kinase receptors are inhibited by the drug, thus interfering with cell proliferation. Furthermore, c-Abl can promote fibrosis as an important downstream target of TGF-β [14]. This leads to the hypothesis that tyrosine kinase inhibition of PDGF receptors and c-Abl by Imatinib represents a single therapy capable of inhibiting activity of two profibrotic growth factors TGF-β and PDGF.

The present study was designed to explore the renoprotective potential of the orally active tyrosine kinase inhibitor Imatinib in a chronic model of progressive mesangioproliferative glomerulonephritis. The major findings are 1) Imatinib remarkably limits the progressive course of chronic anti-thy1 antibody-induced renal disease as shown by functional and morphological estimates; 2) the renoprotective action of Imatinib involved beneficial effects on key pathways of progressive renal disease such as decreased TGF-beta protein expression, matrix protein accumulation, renal cell proliferation, myofibroblast activation and inflammatory cell infiltration; 3) these actions were most prominent in the tubulointersitial compartment and less in the glomerular space. In the following we will discuss the relevance and implications of these findings.

Previous studies have shown that beneficial effects of Imatinib in some models of renal fibrosis, such as acute anti-thy1 glomerulonephritis of the rat [15], lupus nephritis [26,27], hypertensive nephropathy [28], diabetic nephropathy [19], unilateral ureteral obstruction [29], chronic allograft nephropathy [30]. In acute anti-thy1 glomerulonephritis, a rat model of acute, reversible glomerular matrix expansion, it was showed that PDGF receptor tyrosine kinase blockade with STI 571 was associated with significant reductions in mesangial cell proliferation, the number of activated (alpha-smooth muscle positive) mesangial cells, and glomerular type IV collagen deposition. These findings are now expanded into a chronic renal disease model with a distinct injurious glomerular insult in the beginning and subsequent progressive tubulointerstitial fibrosis and renal insufficiency driven by, not primarily immune-mediated, rather autonomously intrarenal mechanisms, which are shared by many other chronic kidney diseases and are in a line with the concept that a common final pathway underlies the advance of renal disease. Compared with the daily intraperitoneal dose 50 mg/kg in the acute anti-thy1 model, Imatinib was given orally in relative low dose 10 mg/kg, which was clinically more relevant und combined with less side effects.

This contrasts to diabetic and hypertensive nephropathy in which extrarenal stimuli, such as high blood pressure or hyperglycaemia damage the kidney continuously and thereby maintain disease progress. The same applies to lupus nephritis and chronic allograft nephropathy, in which the ongoing injurious stimuli are of primary immunologic nature. In this sense, the model of anti-thy1-induced, chronic progressive renal fibrosis may be seen as representation of patients with primary glomerular disease who progress to end-stage renal disease after a single episode of glomerulonephritis. In addition, the findings of this study put a new perspective of the therapeutic mechanism of Imatinib on chronic renal disease.

There is a vast of evidence that TGF-β and PDGF closely and jointly mediate and promote the progression of renal disease. In this study, we found a marked reduction in renal TGF-β1 protein expression by the inhibitory action of Imatinib. There are at least two mechanisms contributing to the reduction of TGF-β. PDGF and TGF-β interact with each other and have overlapping biologic activities. In vitro, the anti-TGF-β neutralizing antibody clearly inhibited the stimulatory effect of PDGF on type IV collagen production and PDGF also stimulated TGF-beta production in human mesangial cells in a dose-dependent manner [31]. It could also be explained by inhibited downstream target of TGF-β, the Bcr-Abl tyrosine kinase, by Imatinib treatment. In experimental bleomycin-mediated lung fibrosis and unilateral obstructive nephropathy models, the treatment of Imatinib reduces the fibrogenesis via inhibiting fibroblast proliferation which is mediated by the c-abl activation through TGF-β [14,29].

Furthermore, the number of α-SMA-positive myofibroblast was reduced by Imatinib treatment in glomeruli and tubulointerstitium. This is associated with inhibition of TGF-β and PDGF through the administration of Imatinib, since both growth factors participate actively in myofibroblast differentiation. In addition, there was a reduction in renal macrophage infiltration with Imatinib. Importance of PDGF isoforms in the development of kidney diseases was confirmed by a number of in vitro experiments, which showed that PDGF may function as a potent chemoattractant for mesangial cells and leukocytes [32,33]. PDGF and TGF-β are mainly produced by infiltrating inflammatory cells under pathological conditions [21,34]. Therefore, treatment of Imatinib decreased macrophage infiltration, which conversely resulted in a decrease in PDGF and TGF-β production within the renal tissue. Both may have contributed to the improvement of renal fibrosis and function. Finally, there was a reduction in renal cell proliferation with Imatinib. Renal cell proliferation precedes extracellular matrix protein expansion in many kidney diseases. Exogenous administration of PDGF isoforms induced in vitro mesangial cells contraction and rapid proliferation [35,36]; and resulted in mild mesangial cell proliferation in normal rats [21]. Interestingly, the administration of Imatinib had no significant effects on the mRNA expression of PDGF isoforms and its receptors in our study. We think Imatinib may interfere mostly with the downstream of PDGF signal transduction through the inhibition of PDGF receptor tyrosine kinase, and hence has no significant effects on upstream mRNA expression. Therefore, this study in chronic anti-thy1 mesangioproliferative glomerulosclerosis proved that the inhibition of tyrosine kinases signalling through Imatinib directly or indirectly interferes with multiple key pathways to slow the progression of chronic renal disease.

In the present study, benefits at the glomerular level were more moderate than in the tubulointerstitium. We think this could be explained by a beginning of Imatinib therapy as late as 7 days after injection of anti-thy1 antibody, when the glomerular injuries were already established. This view is supported by the renoprotective effects on glomerular mesangioproliferation in acute anti-thy1 induced glomerulonephritis when therapy was started as early as 24 hours after anti-thy1 antibody injection [15].

Systolic blood pressure was significantly lower in the Imatinib-treated animals than in the untreated chronic glomerulosclerosis animals in week 20 after disease induction. This may have contributed to the renoprotection of Imatinib treatment. According to its primary pharmacological action, tyrosine kinase inhibitors possess no direct effect on blood pressure. Therefore, it is likely that the lower blood pressure with Imatinib in this study was mediated indirectly through less renal damage and fibrosis.

So far, there have been undertaken different strategies to block TGF-β and PDGF action in various renal disease models. The administration of neutralizing antibodies against PDGF isoforms and its receptors (PDGF-A, -B, -C,-D, PDGF-Rβ) [37-40] and oligonucleotide aptamer antagonist against PDGF [41,42] have already been described. Neutralizing the actions of TGF-beta with either an antibody or the proteoglycan decorin has been shown to prevent excessive matrix accumulation after tissue injury [43,44]. PDGF antagonists mentioned above had a beneficial effect on renal disease in vivo experiments in spontaneously hypertensive rats, model of unilateral ureteral obstruction, streptozotocin-induced diabetes and anti-thy1 induced glomerulonephritis [7]. Compared to other PDGF antagonists with unconvinient application, expensive costs and immunological complications, orally administered Imatinib is well absorbed and has an absolute bioavailability of 98% without high production costs and immunological complications. In this context we would like to point out that Imatinib was even effective in a relative low dose of 10 mg/day/Kg in chronic anti-thy 1 glomeruloslerosis as compared to other renal disease models [15,26-29].

Imatinib, the first generation to be established as c-abl and PDGF receptor inhibitor, is considered standard frontline therapy for the management of patients with chronic myeloid leukemia. However, there has been concern over the emergence of resistance to imatinib, and some patients fail to respond or are intolerant of imatinib therapy because of untoward toxicity. The side effects of Imatinib are dose-dependent and include oedema, muscle cramps, diarrhea, and bone marrow toxicity [45]. Imatinib might also slightly increase the risk of congestive heart failure, especially in patients with a previous history of heart disease [46]. Dasatinib, nilotinib and Bosutinib, the second gerneration inhibitors of c-abl and PDGF receptors, serve as salvage therapies for the treatment of refractory chronic myeloid leukemia as well as patients with intolerance to Imatinib [47,48]. Although these agents are active, third-generation TKIs are under development for patients who either have failed sequential therapy with at least two TKIs or carry the highly resistant T315I mutation. Some of these agents have already shown promising clinical activity. However, longer follow-up is warranted to unveil the potential of these agents in progressive fibrotic changes and their unwanted toxicity.

## Conclusions

PDGF plays a major role in stimulating the replication, survival and migration of myofibroblasts, while TGF-β1 primarily functions in fibrogenesis to stimulate collagen deposition by newly replicated myofibroblasts. In chronic renal disease, both cytokines play a dependently or independently role in disease progression. In a model of chronic anti-thy1-induced mesangioproliferative glomerulosclerosis, we found that administration of Imatinib slows its progressive course toward chronic renal fibrosis and insufficiency. The beneficial effects of Imatinib are associated with improvement in proteinuria, extracelluar matrix protein accumulation, renal myofibroblast differentiation, renal cell proliferation and macrophage infiltration, which are crucial for the progression of chronic renal disease. The renoprotective actions may involve the antagonism of PDGF receptor tyrosine kinase and inhibition of TGF-β mediated by bcr-Abl activation. These findings suggest the tyrosine kinase inhibitors, such as Imatinib, may be an effective approach in slowing the progression of chronic glomerular disease.

## Abbreviations

cGS: chronic glomerulosclerosis; ELISA: Enzyme-linked immunosorbent assay; GFR: Glomerular filtration rate; TGF-β: Transforming growth factor-β; PBS: Phosphate-buffered saline; PCNA: Proliferating-Cell-Nuclear-Antigen; PDGF: Platelet-derived growth factor; SMA: α-smooth muscle actin; TIMP-1: Tissue inhibitor of metalloproteinase-1.

## Competing interests

This study was support be a grant from Novartis Pharma GmbH, Nuremberg, Germany. HP received honoraria for educational courses by Novartis Pharma GmbH.

## Authors’ contributions

YW designed the experiments, analyzed data and wrote the first draft of the manuscript; HP conceived the study and its design, participated in the statistical analyses and interpretation of the data, and writing the manuscript. HK and HHN conceived and designed the experiments. AM, DK and TL performed the experiments. All authors read and approved the final manuscript.

## Pre-publication history

The pre-publication history for this paper can be accessed here:

http://www.biomedcentral.com/1471-2369/14/223/prepub
